# Plasma Cells in the Melanoma Tumor Microenvironment—Mechanistic Roles for IgA

**DOI:** 10.3389/fimmu.2020.00979

**Published:** 2020-06-05

**Authors:** Rakesh Verma, Lalit Kumar

**Affiliations:** ^1^Yale Cancer Center, Yale University, New Haven, CT, United States; ^2^Department of Medical Oncology, Dr. BR Ambedkar Institute Rotary Cancer Hospital, All India Institute of Medical Sciences, New Delhi, India; ^3^All India Institute of Medical Sciences, New Delhi, India

**Keywords:** plasma cells, biomarker, IgA, immune therapies, checkpoint blockade, tumor microenvironment, PD1

Immune check point blockade therapies have evolved to gain major roles in frontline treatment of multiple cancers including melanoma. Recent approvals of therapeutic antibodies that block cytotoxic T lymphocyte associated antigen 4 (CTLA4) and programmed cell death protein 1 (PD1) in melanoma, non-small-cell lung cancer and kidney cancer illustrate the importance of identifying additional immune checkpoints that could be targeted clinically. Our existing knowledge of tumor-microenvironment in melanoma and other cancers remain limited. Enhanced understanding of tumor-microenvironment will bring additional required knowledge base to help optimize use of drugs that block these pathways in tumor microenvironment. Defining biomarkers that predict therapeutic effects and adverse events also require further lead efforts, in addition to existing knowledge about the role of T cells. Bosisio et al. comprehensively reported the pathological features and clinical outcomes of primary cutaneous melanomas associated with plasma cell infiltration (1). Melanoma has been considered one of the most “immunogenic cancers” due to its ability to undergo spontaneous regression and the association of lymphocyte infiltration within areas of histologic regression (2). Over the past few years, much attention has focused on therapeutic applications of T cell immunity in melanoma following the success of immune checkpoint inhibitors. For example, the mechanism by which CTLA-4 and PD-1 blockade mediate T cell activation has been explored by several studies (3–5). However, a substantial knowledge gap still exists with respect to the other arm of the adaptive immune system in this disease.

The function of B cells and plasma cells (PCs) has remained obscure in the melanoma tumor microenvironment. Limited earlier reports (6) illustrated the potential of PCs to be a predictor of inferior outcomes, and Bosisio et al. (1) further demonstrated the underappreciated role for PCs in melanoma prognosis. Plasma cell infiltration correlated with negative prognostic markers (high Breslow thickness, >6 mitoses/mm^2^, ulceration, lymphatic, and vascular invasion). A significant proportion of PCs within primary melanoma tumors and draining lymph nodes expressed a restricted, oligoclonal IgA repertoire (1). Germain et al. also recently reported the presence of IgA at the tumor site as intriguing because it differs from the usual IgG detected in the periphery. This further highlight the need to distinguish between local and systemic differences using repertoire analyses (7). It remains to be seen what specialized characteristics of the tumor microenvironment of this tumor-subset including antigens types drive the plasma cell response. Additionally, monoclonal IgA-s could serve as an ideal starting point from which to begin the search for potential antigens.

Immunosuppressive therapies have been correlated with a higher incidence of melanoma and poorer survival, indicating that melanoma tumor cells may be susceptible to surveillance by the immune system (8). This leads to another fundamental question—do plasma cells actively orchestrate immune evasion through mechanisms yet unknown, or is their presence only a symptom of immunosuppression. Recent studies of global gene expression studies to probe B cell signatures with B cell TILs have reported an association with favorable prognosis across tumor types (9–12). Further in case of metastatic melanoma, B cells in the follicles of tertiary lymphoid structure (TLS) elicited IgA responses (7). For melanoma, the high expression of a B cell signature predicted improved OS, based on mRNA sequencing data analysis (*n* = 329) (13). Recent studies have also suggested the role of *in situ* production of tumor-specific antibodies as triggered by tumor escape mechanisms. Further, if these antibodies contribute to anti- or pro-tumor responses depends on the class of antibodies produced and their glycosylation patterns (14). Prognostic implications of the antibody response can be differential due to their diverse and polyfunctional nature (7).

Additional evaluations are required to reveal a poor response to anti-PD-1/CTLA-4 therapy in patients with plasma cell-rich melanomas. Infiltrating plasma cells on the other hand may have an initial anti-tumor effect but are inactivated by an immunosuppressive milieu. IgA switching may be a consequence of the secretion of transforming growth factor-β (TGF-β) by regulatory T lymphocytes (Figure 1). This offers a possible explanation for patients with thick melanomas, scattered plasma cell infiltration and clustered/sheets of plasma cell infiltration offer paradoxically better and poorer survival than PC-negative patients, respectively. Further studies with additional analysis of the other immune components within the primary tumor and microenvironment will provide greater context to help elucidate the true function of B cells and plasma cells.

**Figure 1:**
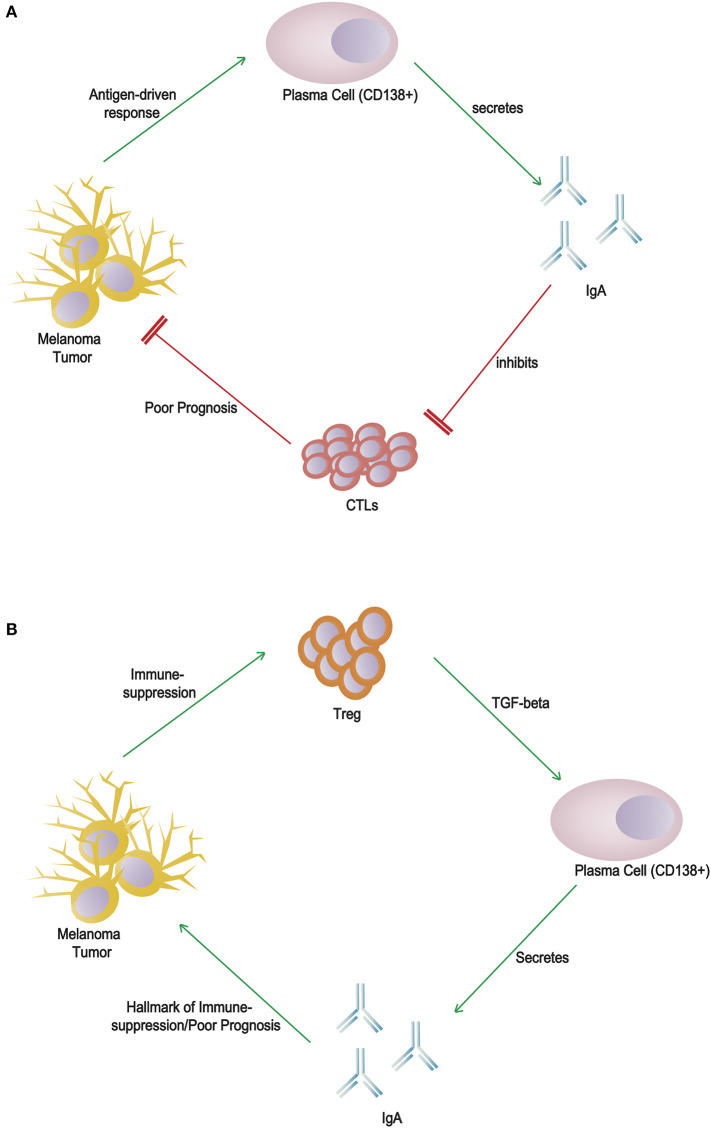
Alternative hypotheses for the role(s) of CD138+ plasma cells and IgA in melanoma tumor microenvironment. **(A)** Direct cytotoxic T lymphocyte inhibition by IgA: an antigen-driven response induces differentiation of plasma cells and secretion of IgA, which directly inhibits cytotoxic T lymphocytes. **(B)** Indirect marker of immunosuppression: regulatory T lymphocytes within the immunosuppressed environment secrete TGF-β, which induces class switching by B cells to produce IgA, a poor prognostic marker.

## Author Contributions

RV prepared the manuscript. RV and LK wrote the manuscript.

## Conflict of Interest

The authors declare that the research was conducted in the absence of any commercial or financial relationships that could be construed as a potential conflict of interest.
